# Fe-Incorporated Nickel-Based Bimetallic Metal–Organic Frameworks for Enhanced Electrochemical Oxygen Evolution

**DOI:** 10.3390/molecules28114366

**Published:** 2023-05-26

**Authors:** Dan Wang, Fuhe Le, Jing Lv, Xue Yang, Xianhao Chen, Haibin Yao, Wei Jia

**Affiliations:** 1 State Key Laboratory of Chemistry and Utilization of Carbon Based Energy Resources, College of Chemistry, Xinjiang University, Urumqi 830017, China; 2 Xinjiang Uygur Autonomous Region Research Institute of Measurement & Testing, Urumqi 830011, China; 3 Quality and Safety Testing Center of Urumqi Agricultural Products, Urumqi 830000, China

**Keywords:** metal–organic frameworks, NiFe-BDC, electrocatalytic oxygen evolution, synergistic effect

## Abstract

Developing cost-effective and high-efficiency catalysts for electrocatalytic oxygen evolution reaction (OER) is crucial for energy conversions. Herein, a series of bimetallic NiFe metal–organic frameworks (NiFe-BDC) were prepared by a simple solvothermal method for alkaline OER. The synergistic effect between Ni and Fe as well as the large specific surface area lead to a high exposure of Ni active sites during the OER. The optimized NiFe-BDC-0.5 exhibits superior OER performances with a small overpotential of 256 mV at a current density of 10 mA cm^−2^ and a low Tafel slope of 45.4 mV dec^−1^, which outperforms commercial RuO_2_ and most of the reported MOF-based catalysts reported in the literature. This work provides a new insight into the design of bimetallic MOFs in the applications of electrolysis.

## 1. Introduction

The development of green, clean, and sustainable energy sources has become increasingly important in view of alleviating environmental pollution caused by fossil fuel combustion [1,2,3,4]. The electrocatalytic oxygen evolution reaction (OER) is a crucial reaction in water splitting and in many renewable-energy-related devices. However, as a typical four-electron transfer process, OER is severely hindered by its intrinsically sluggish kinetics, which requires a large potential to overcome the reaction energy barrier [5,6]. Presently, IrO_2_ and RuO_2_ are the benchmark catalysts for OER. Nevertheless, scarcity, high price, and poor stability have greatly restricted their applications [7,8,9,10,11]. Therefore, to address these drawbacks, tremendous efforts have been devoted to developing Ir/Ru-free catalysts for OER.

In the past few years, earth-abundant and cost-effective transition-metal-based electrocatalysts have been widely investigated as alternatives for noble metals. Among them, Ni-based materials, such as oxides [12,13], hydroxides [6,14], phosphides [15,16], sulfides [17], nitrides [18,19,20], and metal–organic frameworks (MOFs) [21,22] have been extensively studied as alternative catalysts for OER due to their large accessible active sites and good electrical conductivity. As is known, electrocatalysis usually occurs on the surface of the catalysts. Therefore, the OER performance is strongly related to the surface structure of a catalyst. For instance, tailoring the size of a catalyst can significantly increase the surface-to-volume ratio, leading to several attractive features, such as a unique morphology and distinctive electronic structure, and thereby facilitating the catalytic activity [23,24,25,26]. Therefore, the rational design of electrocatalysts with a large surface area and a high exposure of active sites is vital to achieve a high activity. Benefiting from the rich combinations of metal centers and functional ligands as well as a large surface area and porosity, MOFs have drawn particular attention in many fields. Considering that MOFs usually possess low electrical conductivities and electrocatalytic activities, directly using MOFs as electrocatalysts remains a great challenge. Recently, pyrolysis of MOFs has been demonstrated as an effective way to synthesize MOF-derived electrocatalysts, including metal oxides [27], phosphides [28], nitrides [29] etc. However, high-temperature pyrolysis treatment results in a severe aggregation and a sacrifice of organic ligands, which not only collapses the structure, but also reduces the number of active sites. In addition, compared with monometallic MOFs, bimetallic MOFs can provide higher oxidation states of transition metal species and synergistic interactions between different metal ions, which make them more favorable for OER [30,31]. In addition, two-dimensional (2D) MOFs with a high exposure of unsaturated coordinative metal active sites and increased surface areas facilitate mass transport and electron transfer during electrocatalysis [32]. Thus, directly designing bimetallic 2D MOFs as OER catalysts is a promising way to achieve high OER activity. For instance, Jia et al. reported the use of 2D ultrathin NiFe-UMNs for OER [33]. The abundant coordinatively unsaturated Ni active sites, enhanced e_g_-orbital filling and higher valence of Ni ions make the NiFe-UMNs exhibit excellent electrocatalytic performances in basic media. Zhuang and co-workers verified that the synergistic interaction between Co and Fe ions endows the FeCo-MOFs nanosheets with outstanding OER performances [34]. Although significant progresses have been made, in-depth investigations on functional MOF materials that can be directly employed as OER catalysts are highly required.

Herein, 1,4-bezenedicarboxylate (1,4-BDC) was used as an organic ligand to prepare NiFe bimetallic MOF (NiFe-BDC-0.5) nanosheets via a facile solvothermal treatment. Compared with the monometallic Ni-BDC, the appropriate introduction of Fe into the Ni-BDC leads to partial charge transfer from Ni ions to Fe ions, increasing the oxidation state of Ni ions, thereby boosting the activity of OER. Furthermore, an electrochemical reconstruction occurs during OER, which leads to the generation of NiFe (oxy)hydroxide and more Ni sites serving as the electrochemical active sites, endowing the NiFe-BDC-0.5 with improved OER performances. Specifically, the NiFe-BDC-0.5 exhibits a low overpotential of 256 mV to achieve a current density of 10 mA cm^−2^ and a long-term stability over 50 h in 1 M KOH, which is superior to that of RuO_2_ and most of the reported transition-metal-based catalysts reported in the literature.

## 2. Results and Discussion

**Figure 1:**
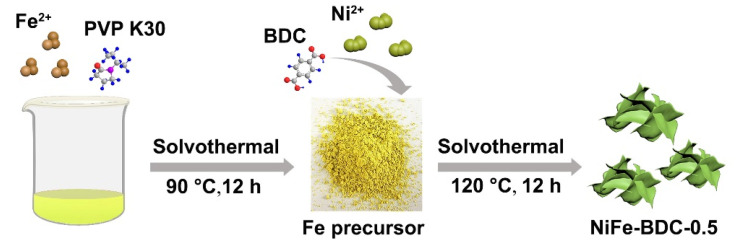
Schematic illustration of the preparation of the NiFe-BDC-0.5.

**Figure 2:**
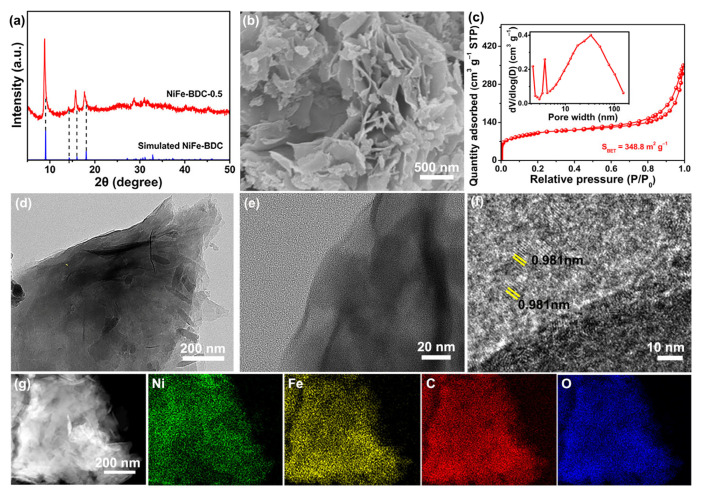
Structure and morphology of NiFe–BDC–0.5. (**a**) XRD patterns, (**b**) SEM image, (**c**) N_2_–adsorption/desorption isotherms and pore size distribution, (**d**) TEM image, (**e**) enlarged TEM image, (**f**) HRTEM image, (**g**) HAADF–STEM and corresponding elemental mappings.

A series of bimetallic metal–organic frameworks NiFe-BDC-x (x = 0.1, 0.3, 0.5, 0.7) were synthesized by a simple solvothermal method. Among the as-prepared samples, the NiFe-BDC-0.5 exhibits the highest activity toward OER; therefore, the NiFe-BDC-0.5 is primarily discussed in this work. The preparation route of the bimetallic metal–organic frameworks (NiFe-BDC-0.5 MOF) is illustrated in Figure 1. First, an Fe-based metal–organic compound (Fe-precursor) was prepared via a solvothermal reaction. The power X-ray diffraction (XRD) pattern of the Fe-precursor (Figure S1) is consistent with that reported in the literature [35], indicating the successful synthesis of the Fe-precursor. Subsequently, the NiFe-BDC-0.5 nanosheets are obtained by adding NiCl_2_·6H_2_O to react with the Fe precursor through a simple solvothermal treatment. By analyzing the phase of the NiFe-BDC-0.5, it can be observed that the main diffraction peaks centered at 8.8°, 14.2°, 15.7°, and 17.7° in the XRD pattern (Figure 2a) can be attributed to the (200), (001), (201), and (400) crystal planes of MIL-53(NiFe) (CCDC No. 985792), suggesting that the Ni-MOF was successfully prepared [36,37]. The XRD patterns of the as-prepared NiFe-BDC-x with different added amounts of Ni are displayed in Figure S2. It is obvious that the intensities of the diffraction peaks increase and become sharper with an increase in the addition of Ni in NiFe-BDC. Meanwhile, the diffraction peaks of the NiFe-BDC-x (x = 0.1, 0.3, 0.5, 0.7) shift slightly toward lower angles compared with those of the simulated MIL-53(NiFe). In addition, the XRD pattern of the Ni-BDC, which was prepared without the addition of Fe-precursor, is also similar to that of NiFe-BDC-0.5 (Figure S3). All these points indicate the successful preparation of NiFe bimetallic MOFs.

The morphologies and structures of the Ni-BDC and NiFe-BDC-x were observed using a scanning electron spectroscope (SEM) and transmission electron microscope (TEM). As illustrated in Figure S4a, the Ni-BDC displays a sheet-like morphology. When Fe-precursor is added during the preparation process, the morphologies of the as-synthesized samples are closely related to the added amount of NiCl_2_·6H_2_O. When adding 0.1 mmol of NiCl_2_·6H_2_O, the as-obtained NiFe-BDC-0.1 consists of nanoparticles with a size of ~50 nm (Figure S4b). As the added amount of NiCl_2_·6H_2_O increases to 0.3 mmol, some nanosheets appear in the NiFe-BDC-0.3 (Figure S4c). Upon further increasing the amount of NiCl_2_·6H_2_O to 0.5 mmol, the morphology of the NiFe-BDC-0.5 is basically identical to that of the Ni-BDC (Figure 2b). When the molar ratio of Ni^2+^ to BDC is lower than 1:1, the coordination between Ni^2+^ ions and BDC ligands is insufficient, and the BDC ligands cannot be connected to form the sheet-like structure. When the ratio is higher than 1:1, the nanosheets pack together, resulting in an aggregated morphology and a low exposure of active sites (Figure S4d). By carefully comparing the SEM images of Ni-BDC and NiFe-BDC-0.5, it can be seen that the thickness of the NiFe-BDC-0.5 nanosheet is thinner than that of the Ni-BDC, implying that the introduction of Fe-precursor can reduce the thickness of the nanosheets and increase the specific surface area. The N_2_ adsorption–desorption isotherm of NiFe-BDC-0.5 is displayed in Figure 2c. It is clear that the NiFe-BDC-0.5 exhibits a type IVa adsorption isotherm with H3-type hysteresis loops, which is commonly found in layered structures. The specific surface area of the NiFe-BDC-0.5, based on the Brunauer–Emmett–Teller (BET) method, is 344.8 m^2^ g^−1^, and the pore size is mainly distributed in a range of 30–40 nm, which indicates its mesoporous structure. The unique nanosheets and mesoporous feature are favorable for an increase in the number of active sites and promote mass transfer, and thus improve the OER performance. The transmission electron microscopy (TEM) image of the NiFe-BDC-0.5 further displays that the ultrathin nanosheets are hundreds of nanometers in size, and curling can be observed at the edges (Figure 2d,e), further demonstrating its ultrathin nature. The high-resolution TEM (HRTEM) image (Figure 2f) reveals that these nanosheets are composed by amorphous and polycrystalline regions. The lattice distance of 0.981 nm in the crystalline region is close to the theoretical values of the (200) plane [38]. The elemental mapping images shown in Figure 2g demonstrate a uniform distribution of Ni, Fe, C, and O elements over the entire NiFe-BDC-0.5. To further reveal the composition of the as-obtained samples, energy-dispersive X-ray spectroscopy (EDX) was then carried out (Figure S5), which confirmed the existence of Ni, Fe, C, and O elements in the four samples. The atomic ratios of Ni to Fe in the NiFe-BDC-x were determined to be 0.5, 1.5, 2.5, and 3.6, respectively, which increased with the added amount of NiCl_2_·6H_2_O.

**Figure 3:**
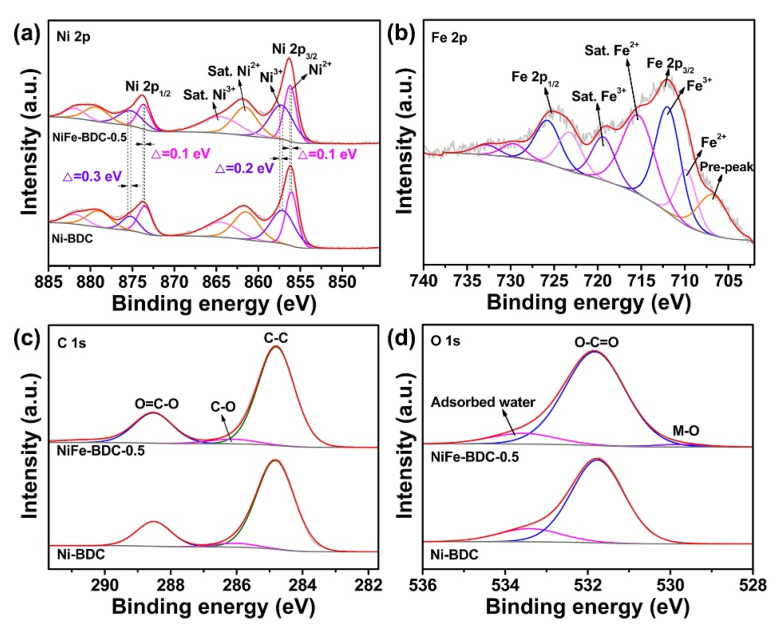
XPS spectra of (**a**) Ni 2p for the NiFe-BDC-0.5 and Ni-BDC, (**b**) Fe 2p of the NiFe-BDC-0.5, (**c**) C 1s, and (**d**) O 1s for the NiFe-BDC-0.5 and Ni-BDC.

The chemical valences of the Ni-BDC and NiFe-BDC-0.5 were then investigated by X-ray photoelectron spectroscopy (XPS). The XPS survey scan shows the presence of Ni, Fe, C, and O elements in the NiFe-BDC-0.5 (Figure S6). The deconvoluted results of Ni 2p spectra are presented in Figure 3a. For the NiFe-BDC-0.5, peaks at 856.2 eV and 873.7 eV can be assigned to the Ni^2+^ 2p_3/2_ and Ni^2+^ 2p_1/2_, respectively. The corresponding satellite peaks are centered at 861.4 eV and 879.2 eV. The existence of Ni^2+^ originated from the link between Ni^2+^ and BDC ligands [39,40]. In addition, peaks located at 857.4 eV (Ni^3+^ 2p_3/2_) and 875.3 eV (Ni^3+^ 2p_1/2_) along with two satellite peaks at 864.4 eV and 881.8 eV indicate the existence of Ni-OH [41,42]. The relative peak areas for Ni^3+^ 2p and Ni^2+^ 2p show that the Ni species exist as 45% of Ni^2+^ and 55% of Ni^3+^. In the Fe 2p spectrum displayed in Figure 3b, the binding energies located at 709.8 eV (Fe^2+^ 2p_3/2_), 723.2 (Fe^2+^ 2p_1/2_), 711.9 eV (Fe^3+^ 2p_3/2_), and 725.7 eV (Fe^3+^ 2p_1/2_) demonstrate that Fe mainly exists as Fe^3+^ and Fe^2+^ species [43,44]. Additionally, compared with the Ni-BDC, the peaks of Ni 2p in NiFe-BDC shift toward higher binding energies, suggesting the increase in the Ni valence after Fe introduction due to a synergistic interaction between Ni and Fe ions. Theoretically, the valence electronic configuration of Ni^2+^ is 3d^8^ and the corresponding orbital occupancy is t_2g_^6^e_g_^2^ [45], while the valence electronic configuration of Fe^3+^ is 3d^5^ and the e_g_ orbital of Fe^3+^ (3d^5^, t_2g_^5^e_g_^0^) is unoccupied [34]. As previously reported [38,46], when Ni^2+^ is coupled with Fe^3+^, the charge can be partially transferred from Ni^2+^ to Fe^3+^ and can generate more high-valence Ni ions. The higher oxidation state of Ni ions is favorable for increasing the adsorption of OH^−^, which can significantly boost the charge transfer between NiFe-BDC and OH^−^, thereby optimizing the OER performance [30,47]. Figure 3c depicts the C 1s spectra of Ni-BDC and NiFe-BDC, which can be fitted into three characteristic peaks at 284.8 eV, 286.1 eV, and 288.5 eV, belonging to the C=C/C-C bonds, C-O bond, and the carboxyl (O-C=O) groups in BDC ligands, respectively [38]. The XPS spectra of O 1s can be deconvoluted into three peaks at 529.8 eV, 531.8 eV, and 533.8 eV, which originate from oxygen atoms on the Ni (Fe)-O, O-C=O, and absorbed water [48]. The above XPS analyses further demonstrate the coordination between Ni^2+^/Fe^3+^ ions and BDC ligands, which is consistent with the XRD results.

**Figure 4:**
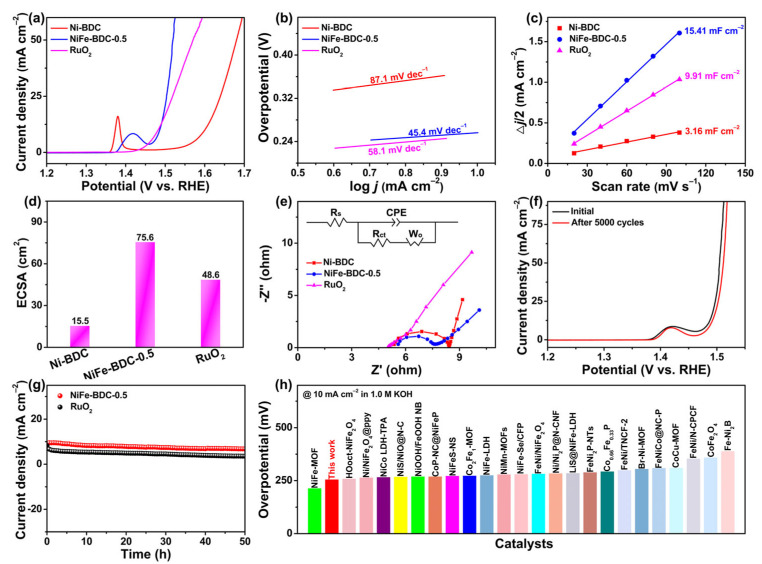
Electrocatalytic OER performance of the NiFe-BDC-0.5 and other comparable samples tested in 1 M KOH solution. (**a**) LSV curves, (**b**) Tafel plots, (**c**) C_dl_, (**d**) ECSA, and (**e**) EIS of the Ni-BDC, NiFe-BDC-0.5, and RuO_2_. (**f**) Polarization curves of the NiFe-BDC-0.5 before and after 5000 CV cycles. (**g**) Time-dependent current density curves of the NiFe-BDC-0.5 and RuO_2_ under a constant overpotential of η_10_ for 50 h. (**h**) Comparisons of OER activity of the NiFe-BDC-0.5 with recently reported MOF-based electrocatalysts.

The electrocatalytic OER performances of the NiFe-BDC-0.5 was studied in 1 M KOH. The linear sweep voltammetry (LSV) curve of the NiFe-BDC-0.5 was compared with those of Ni-BDC and RuO_2_. As depicted in Figure 4a, the Ni-BDC exhibits unsatisfactory OER activity with an overpotential of 371 mV at a current density of 10 mA cm^−2^. With the introduction of Fe into the Ni-BDC, the OER activity of the NiFe-BDC-0.5 increases remarkably, which only needs an overpotential of 256 mV to achieve 10 mA cm^−2^. Impressively, the activity of the NiFe-BDC-0.5 surpasses commercial RuO_2_ when the current density exceeds 13 mA cm^−2^. Furthermore, pronounced oxidation peaks centered at ~1.38 V were observed in the LSV curves of the Ni-BDC and NiFe-BDC-0.5, which can be ascribed to the transformation of Ni^2+^ to NiOOH [49,50]. Earlier studies have verified that the NiOOH is more active than Ni^2+^ species toward OER in Ni-based electrocatalysts [51,52,53]. Meanwhile, the oxidation peak of Ni^2+^ to NiOOH shifts to a higher potential (~1.42 V) and the integral area of the peak is enhanced by 66% with the addition of Fe, suggesting the generation of a more active NiOOH phase by introducing Fe into the Ni-BDC. The OER kinetics were then analyzed by the corresponding Tafel slopes (Figure 4b). It is obvious that the Tafel slope of the NiFe-BDC-0.5 is 45.4 mV dec^−1^, which is significantly lower than that of the Ni-BDC (87.1 mV dec^−1^) and RuO_2_ (58.1 mV dec^−1^), implying that NiFe-BDC-0.5 possesses fast OER kinetics and a high affinity toward adsorbed OH^−^ intermediates. Thus, the equilibrium of adsorbed OH^−^ and derived-O-containing intermediates in the following steps can be developed quickly, and therefore the rate-determining step of the OER occurring on the NiFe-BDC-0.5 is the electron-transfer reaction [54,55]. In addition, LSV curves of different NiFe-BDC-x catalysts were compared to investigate the effect of the Ni amount on the OER performance. As shown in Figure S7, all the bimetallic NiFe-BDC-x catalysts exhibit improved OER activities compared with the monometallic Ni-BDC. Among all the as-prepared catalysts, the NiFe-BDC-0.5 exhibits the highest activity and the fastest OER kinetics.

The double-layer capacitance (C_dl_) was determined by cyclic voltammetry (CV) in a 0.1 V potential range without faradaic currents to obtain the electrochemical active area (ECSA) of the catalysts (Figure 4c,d and Figure S8). The value of C_dl_ for NiFe-BDC-0.5 was 15.41 mF cm^−2^, outperforming those of the Ni-BDC (3.16 mF cm^−2^), NiFe-BDC-0.1 (2.83 mF cm^−2^), NiFe-BDC-0.3 (4.09 mF cm^−2^), NiFe-BDC-0.7 (5.02 mF cm^−2^), and RuO_2_ (9.91 mF cm^−2^). This indicates that the unique nanosheet can afford the NiFe-BDC-0.5 the largest electrochemically active surface area (ECSA). In order to obtain an insight into the OER kinetics of the catalysts, electrochemical impedance spectroscopy (EIS) was performed. The Nyquist plots of Ni-BDC, NiFe-BDC-0.5 and RuO_2_ are shown in Figure 4e and the insert is an equivalent circuit. It is clear that the NiFe-BDC-0.5 possesses the smallest charge transfer resistance (R_ct_) of 1.5 Ω, indicating a high charge transfer ability and the fastest OER kinetics [56]. In addition, the NiFe-BDC-x catalysts with different incorporated amounts of Ni were also tested. As expected, the value of R_ct_ follows the same trend as that of the C_dl_, namely NiFe-BDC-0.1 (5.1 Ω) > NiFe-BDC-0.3 (10.9 Ω) > NiFe-BDC-0.7 (3.8 Ω) > NiFe-BDC-0.5 (1.5 Ω) (Figure S9). Apart from activity, long-term operational stability is another essential parameter to evaluate the practicality for electrocatalysts. As shown in Figure 4f, the polarization curve of the NiFe-BDC-0.5 shows a negligible change after uninterrupted 5000 CV scanning compared with the initial one. Figure 4g displays the chronoamperometric curves for the NiFe-BDC-0.5 and RuO_2_ at a fixed overpotential of η_10_. Apparently, the current on the RuO_2_ electrode decreased gradually by 56.7% after 50 h of testing. In contrast, the NiFe-BDC-0.5 only showed 28.0% activity loss, implying a higher stability of the NiFe-BDC-0.5. The superior OER performances of the NiFe-BDC-0.5 may be due to the large electrochemical active area and more active Ni sites, as well as a fast charge transfer. Importantly, the activity of the NiFe-BDC-0.5 outperforms most of the recently reported transition-metal-based MOFs toward the OER (Figure 4h and Table S1).

**Figure 5:**
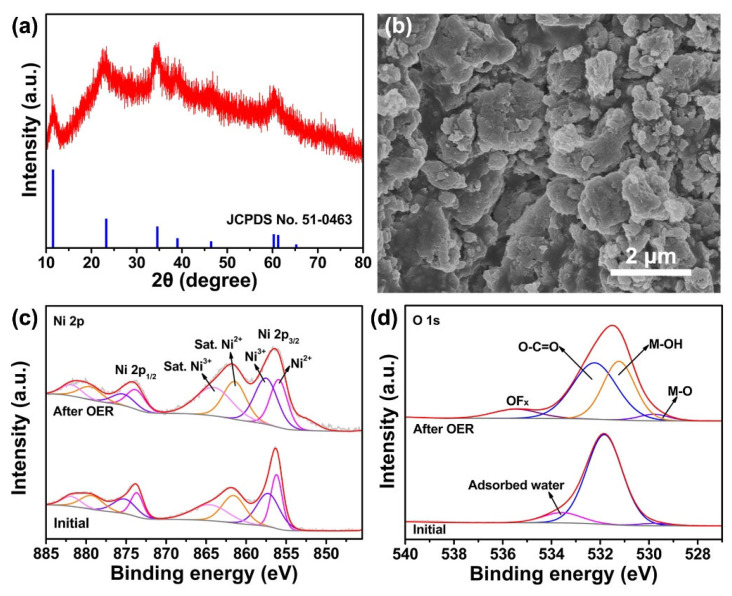
Structural and compositional analysis of the NiFe-BDC-0.5 after OER: (**a**) XRD patterns, (**b**) SEM image, XPS spectra of (**c**) Ni 2p and (**d**) O 1s.

In order to gain a deep insight into the structural evolution of the NiFe-BDC-0.5 during the OER, the phase of the NiFe-BDC-0.5 after the OER was first studied. Figure 5a depicts the XRD pattern of the NiFe-BDC-0.5 after 50 h of testing; it can be seen that the NiFe-BDC-0.5 has completely evolved into NiFe hydroxide carbonate hydrate (JCPDS no. 51-0463). This phenomenon is caused by the organic ligands being replaced by the hydroxide ions and the oxidation of ligands, which usually occurs when MOFs directly electrocatalyze OER in alkaline media [56,57]. The SEM image displayed in Figure 5b reveals that the sheet-like NiFe-BDC-0.5 changes into bulks after the OER. The EDX spectrum illustrates that the contents of Ni and Fe increase from 2.98 at.% and 1.20 at.% to 10.98 at.% and 4.20 at.%, respectively, while C decreases by 17.75 at.% after the OER (Figure S10 and Table S2), indicating that more active Ni species were generated. XPS was then carried out to investigate the change in electronic valence of the NiFe-BDC-0.5 after the OER (Figure S11). The signal F comes from Nafion which was used during the preparation of the electrode. The XPS peaks representing Ni 2p and Fe 2p shift toward higher binding energies and the ratio of Ni^3+^ to Ni^2+^ increases (Figure 5c and Figure S12), verifying that the contents of Ni^3+^ species increase after OER. In addition, the O 1s spectrum of the NiFe-BDC-0.5 after the OER shows the presence of M-O (529.8 eV) and M-OH (531.2 eV), confirming the generation of NiFe hydroxyl oxides, which is consistent with earlier reports [58,59]. The formation of metal-hydroxyl groups (M-OH) significantly improves the hydrophilicity of the catalysts and facilitates the adsorption of H_2_O and –OH groups, and thereby promote oxygen production in the NiFe-BDC-0.5. Based on the above analyses, it can be concluded that the outstanding OER performances of the NiFe-BDC-0.5 originated from its large specific surface area, which exposes more active sites and facilitates the formation of NiFe hydroxyl oxides via structural reconstruction, which facilitates the OER process.

## 3. Materials and Methods

### 3.1. Materials

Nickel chloride hexahydrate (NiCl_2_·6H_2_O), ethanol (CH_3_CH_2_OH), ethylene glycol (C_2_H_6_O_2_), N,N-dimethylformamide (C_3_H_7_NO, DMF), polyvinylpyrrolidone (PVP, K30) and potassium hydroxide (KOH, ≥85%) were purchased from Sinopharm Chemical Reagent Co., Ltd. (Shanghai, China). Iron(II) acetate ([Fe(OAc)_2_]), triethylamine (C_6_H_15_N), and methanol (CH_3_OH) were purchased from Aladdin. Terephthalic acid (C_8_H_6_O_4_, H_2_BDC) and ruthenium (Ⅳ) oxide (RuO_2_) were purchased from Alfa Aesar. Nafion perfluorinated resin solution (5 wt.%) was purchased from Sigma-Aldrich. All chemicals were directly used without further purification.

### 3.2. Synthesis of Fe-Precursor

Fe-precursor was prepared according to a reported method [33]. Typically, 1.15 mmol of Fe(OAc)_2_ and 0.4 g of PVP were dissolved in 5 mL of ethylene glycol under stirring for about 10 min. Then, 25 mL of methanol was poured into the above mixture, and stirred at room temperature for 20 min to obtain a transparent solution. Afterwards, the solution was transferred into a 50 mL Teflon-lined autoclave, following by a solvothermal treatment at 90 °C for 12 h. Finally, the precipitates were washed three times with absolute ethanol and deionized water, and dried in vacuum at 60 °C for 12 h to obtain the Fe-precursor.

### 3.3. Synthesis of NiFe-BDC

The bimetallic NiFe-terephthalic acid (NiFe-BDC-0.5) nanosheets were synthesized by a solvothermal method. First, 0.5 mmol of NiCl_2_·6H_2_O was dissolved in 9 mL of DMF and 1 mL of deionized water under continuous stirring, and 0.5 mmol of H_2_BDC and 300 μL of triethylamine were added in sequence to form the transparent solution. Subsequently, 2 mL of ethanol containing 60 mg of the as-prepared Fe-precursor was added to the above solution. After continuously stirring at room temperature for 30 min, the mixture was transferred into a 20 mL Teflon-lined autoclave, followed by a solvothermal treatment at 90 °C for 12 h. Finally, after washing with ethanol three times and drying in a vacuum at 60 °C for 12 h, the sample was obtained and denoted as NiFe-BDC-0.5. In order to investigate the effect of the Ni content on the OER performance, the NiFe-BDC-x (x = 0.1, 0.3, and 0.7) were also prepared by the same method, except that the added amount of NiCl_2_·6H_2_O was changed from 0.5 mmol to 0.1 mmol, 0.3 mmol, and 0.7 mmol, respectively.

### 3.4. Synthesis of Ni-BDC

Ni-BDC was prepared through the same synthesis procedure as NiFe-BDC except that Fe-precursor was not added.

### 3.5. Material Characterization

X-ray diffraction (XRD) patterns were collected on a Bruker D8 Advance diffractometer equipped with a Cu Kα radiation (λ = 1.5406 Å, 40 kV, 40 mA) ranging from 5° to 80°. Scanning electron spectroscopy (SEM) images and energy-dispersive X-ray spectroscopy (EDX) spectra were collected on a Hitachi SU8010. High-resolution transmission electron microscopy (HRTEM) images and elemental mapping images were obtained on a JEOL JEM-2100F under an accelerating voltage of 200 kV. N_2_-adsorption/desorption isotherms were obtained on a Micromeritics ASAP 2020. The specific surface area and pore size distributions were determined by the Brunauer–Emmett–Teller (BET) method and Density Functional Theory (DFT) algorithm, respectively. X-ray photoelectron spectroscopy (XPS) spectra were collected on a Thermo Fisher Scientific ESCALAB 250Xi instrument, taking C 1s of 284.8 eV as a reference.

### 3.6. Electrochemical Measurements

Electrochemical measurements were performed on a CHI 760E electrochemical workstation (CH Instruments, Inc., Shanghai) with a typical three-electrode system in 1 M KOH solution. A glassy carbon electrode (GCE) with a diameter of 5 mm, an Hg/HgO (filled with 1 M KOH, pH = 14) electrode, and a graphite rod were used as the working electrode, reference electrode, and counter electrode, respectively. To prepare the working electrode, an ink containing 2.5 mg of the catalyst, 480 μL of ethanol and 20 μL of Nafion solution (5 wt.%) was ultrasonically mixed for about 30 min. Then, 10 μL of the well-dispersed ink was dropped onto the surface of the GCE with a fixed catalyst loading of 0.255 mg cm^−2^, then naturally dried in air before the electrochemical test. All the potentials were referenced with a reversible hydrogen electrode (RHE) according to the follow equation:

E_RHE_ = E_Hg/HgO_ + 0.098 V + 0.059 pH

Linear sweep voltammetry (LSV) polarization curves were performed at a scan rate of 5 mV s^–1^. Nyquist plots of electrochemical impedance spectroscopy (EIS) measurements with a frequency ranging from 100 kHz to 0.01 Hz at an amplitude of 5 mV were charted. The electrical double-layer capacitances (C_dl_) were obtained by cyclic voltammetry (CV) curves in a non-faradaic region with 0.1 V potential range. The electrochemically active surface area (ECSA) was obtained by dividing the calculated capacitance to a specific capacitance (C_S_ = 40 μF cm^−2^ in 1 M KOH). Accelerated CV scanning was conducted from 1.1 V to 1.7 V with a scan rate of 100 mV s^−1^ for 5000 cycles to evaluate durability. The long-term stability test was carried out by a chronoamperometric measurement at an overpotential of η_10_ for 50 h. All LSV curves were obtained with 100% *i*R compensation.

## 4. Conclusions

In summary, NiFe bimetallic MOFs (NiFe-BDC-0.5) were prepared by a facile solvothermal method. When directly applied as an OER electrocatalyst, the NiFe-BDC-0.5 transforms into nickel iron hydroxide and more Ni^3+^ active sites are generated. The large specific surface area, synergistic effect between Ni and Fe, as well as the enhanced Ni^3+^ active sites via electrochemical reconstruction ensure a superior OER performance over long-term electrocatalysis in alkaline media, even exceeding commercial RuO_2_ and most of the reported transition-metal-based MOFs. This work provides a simple method for the design of low-cost and highly efficient electrocatalysts based on MOFs.

## Data Availability

Data is contained within the article.

## Supplementary Materials

The following supporting information can be downloaded at: https://www.mdpi.com/article/10.3390/molecules28114366/s1, Figure S1: XRD pattern of the Fe-precursor; Figure S2: XRD patterns of the NiFe-BDC-x (x = 0.1, 0.3, 0.5, 0.7); Figure S3: XRD patterns of Ni-BDC and simulated NiFe-BDC; Figure S4: SEM images of (**a**) Ni-BDC, (**b**) NiFe-BDC-0.1, (**c**) NiFe-BDC-0.3, and (**d**) NiFe-BDC-0.7; Figure S5: EDX spectra of NiFe-BDC-x (x = 0.1, 0.3, 0.5, and 0.7); Figure S6: XPS survey spectra of (**a**) Ni-BDC and (**b**) NiFe-BDC-0.5; Figure S7: (**a**) LSV curves and (**b**) Tafel slopes of the NiFe-BDC-x (x = 0.1, 0.3, 0.5, and 0.7); Figure S8: (**a**–**d**) CV curves, (**e**) C_dl_, and (**f**) ECSA of the NiFe-BDC-x (x = 0.1, 0.3, 0.5, and 0.7); Figure S9: EIS of the NiFe-BDC-x (x = 0.1, 0.3, 0.5, and 0.7); Figure S10: EDX spectrum of the NiFe-BDC-0.5 after OER; Figure S11: XPS survey scan of the NiFe-BDC-0.5 after the OER; Figure S12: Fe 2p XPS spectrum of the NiFe-BDC-0.5 after OER; Table S1: Comparisons of the OER performances of the NiFe-BDC-0.5 with other reported transition-metal-based MOFs in the literature in 1 M KOH [27,28,29,60,61,62,63,64,65,66,67,68,69,70,71,72,73,74,75,76,77,78,79,80]; Table S2: The contents of Ni, Fe, C, and O in NiFe-BDC-0.5 before and after the OER test.

## Sample Availability

Samples of the compounds are available from the authors.
