# Supplementary material for: Fe-Incorporated Nickel-Based Bimetallic Metal–Organic Frameworks for Enhanced Electrochemical Oxygen Evolution
Source: Molecules. 2023 May 26;28(11):4366. doi: 10.3390/molecules28114366 (PMC10254281; doi:10.3390/molecules28114366)
Supplement: Supplementary file 1 [file molecules-28-04366-s001.zip › molecules-2407335-supplementary.pdf]

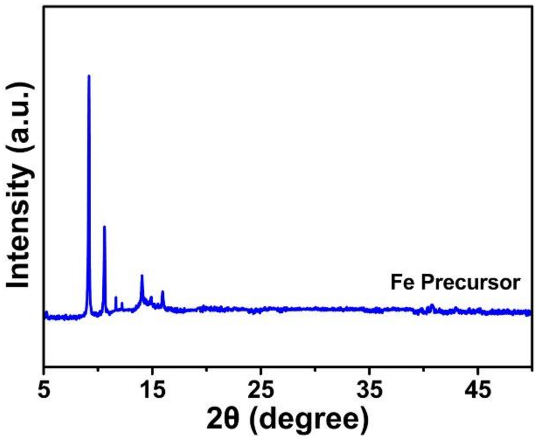

Figure S1. XRD pattern of the Fe-precursor.

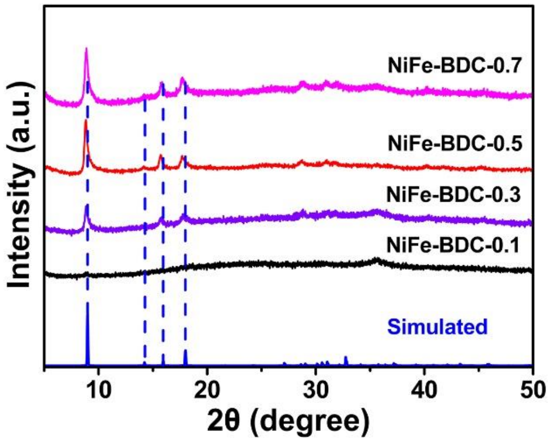

Figure S2. XRD patterns of the NiFe-BDC-x (x = 0.1, 0.3, 0.5, 0.7).

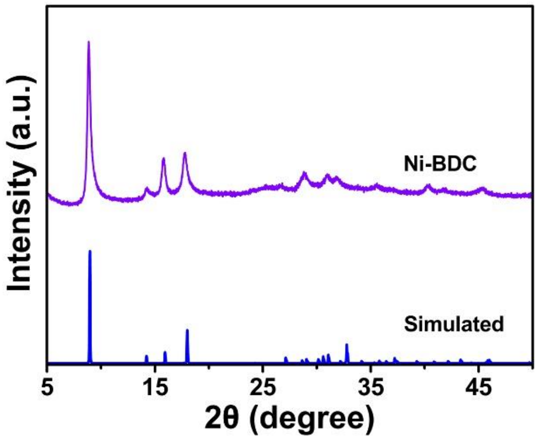

Figure S3. XRD patterns of Ni-BDC and simulated NiFe-BDC.

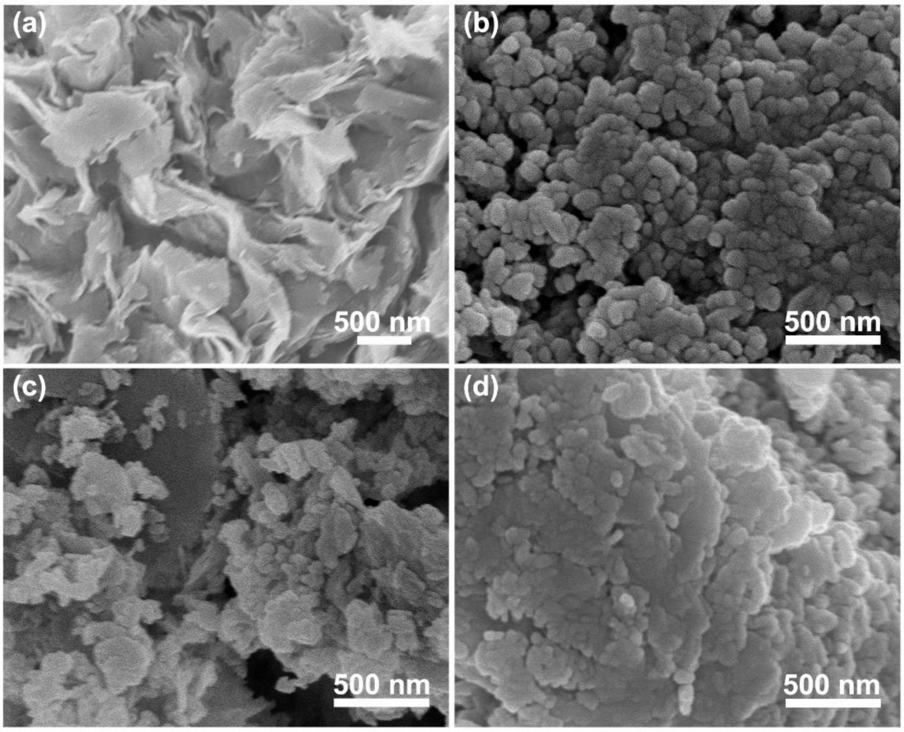

Figure S4. SEM images of (**a**) Ni-BDC, (**b**) NiFe-BDC-0.1, (**c**) NiFe-BDC-0.3, and (**d**) NiFe-BDC-0.7.

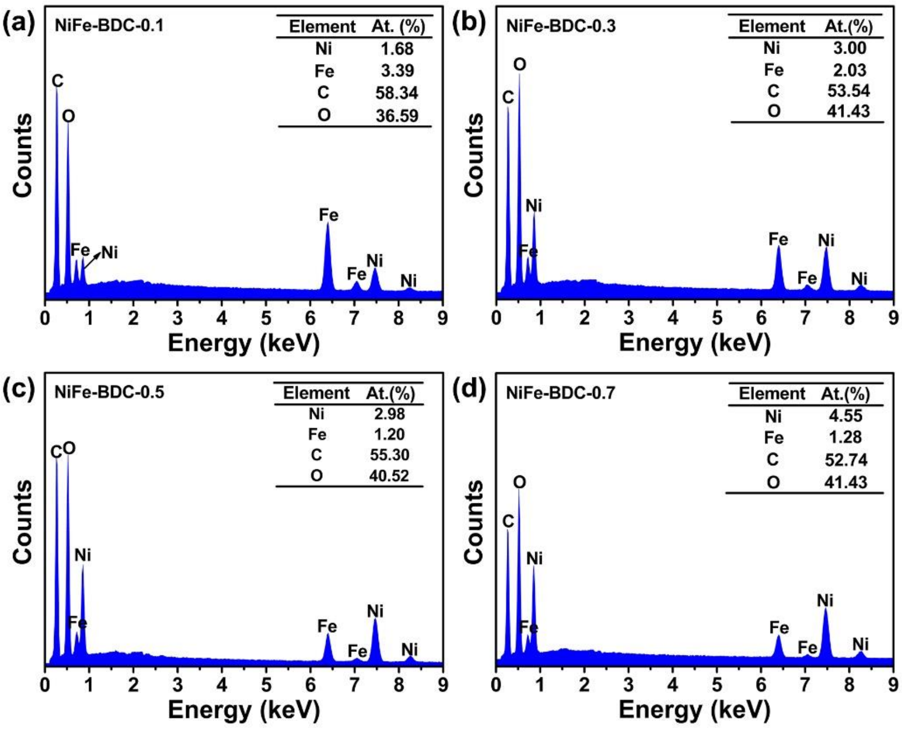

Figure S5. EDX spectra of NiFe-BDC-x (x = 0.1, 0.3, 0.5, and 0.7).

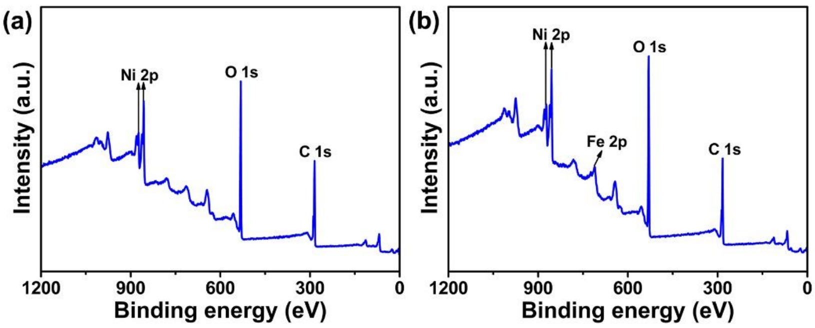

Figure S6. XPS survey spectra of (**a**) Ni-BDC and (**b**) NiFe-BDC-0.5.

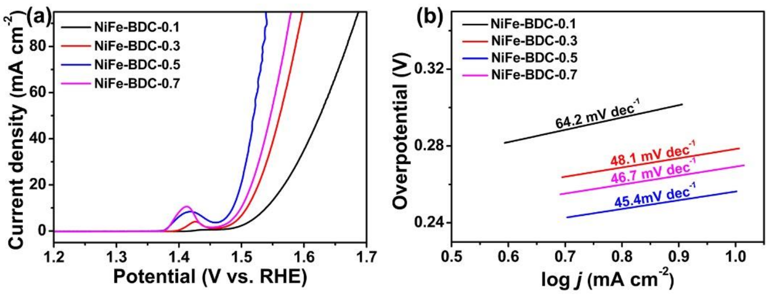

Figure S7. (**a**) LSV curves and (**b**) Tafel slopes of the NiFe-BDC-x (x = 0.1, 0.3, 0.5, and 0.7).

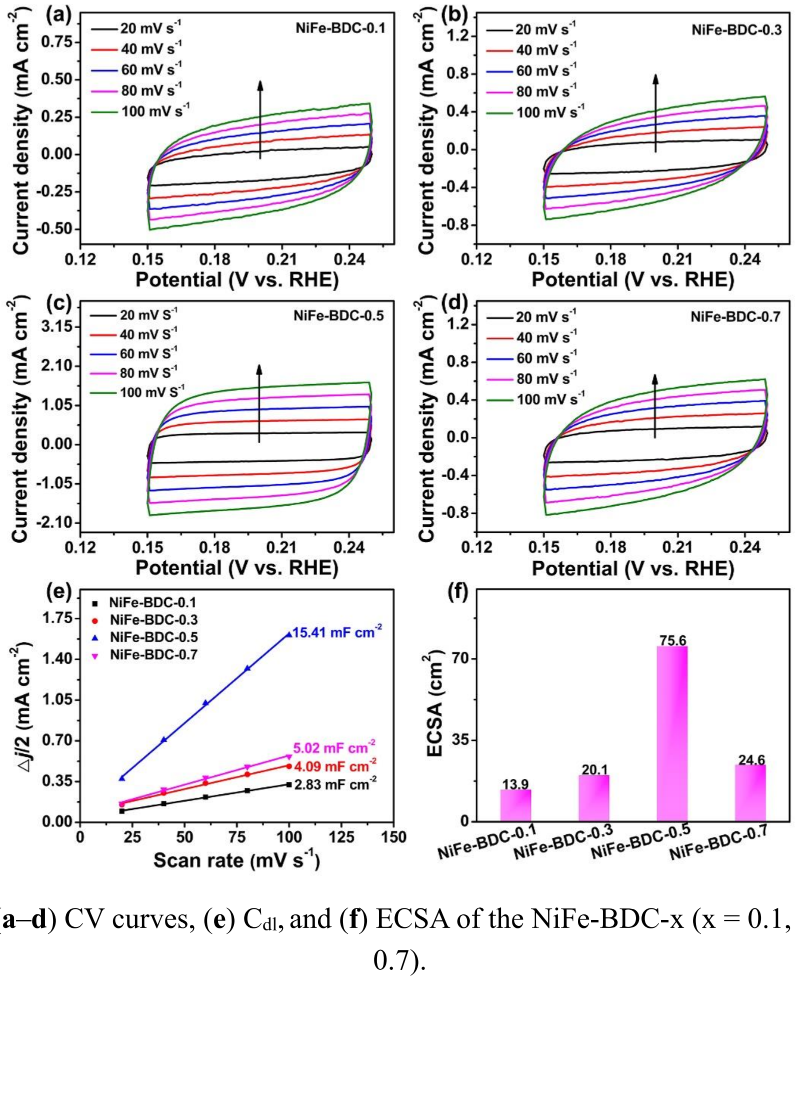

Figure S8. (**a**–**d**) CV curves, (**e**) C_{dl}, and (**f**) ECSA of the NiFe-BDC-x (x = 0.1, 0.3, 0.5, and 0.7).

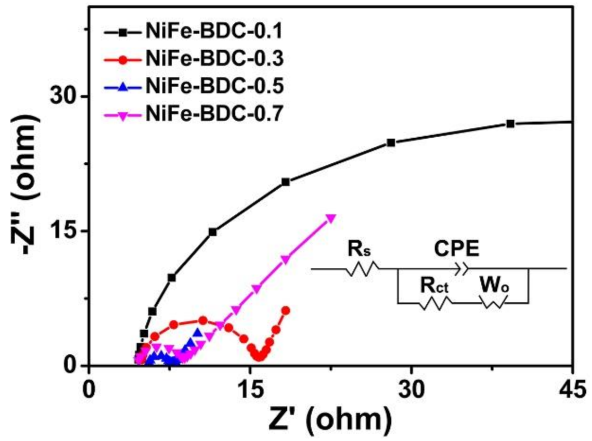

Figure S9. EIS of the NiFe-BDC-x (x = 0.1, 0.3, 0.5, and 0.7).

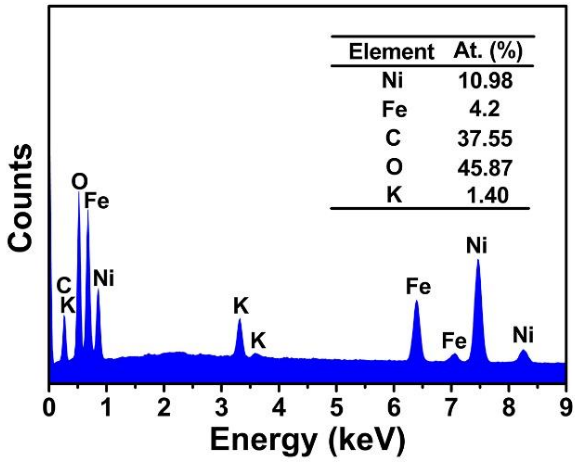

Figure S10. EDX spectrum of the NiFe-BDC-0.5 after OER.

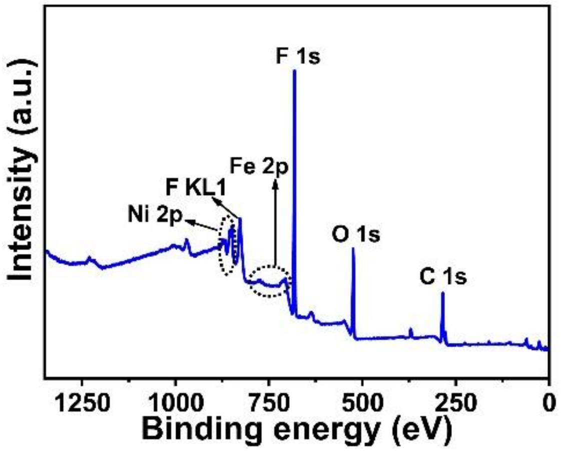

Figure S11. XPS survey scan of the NiFe-BDC-0.5 after OER.

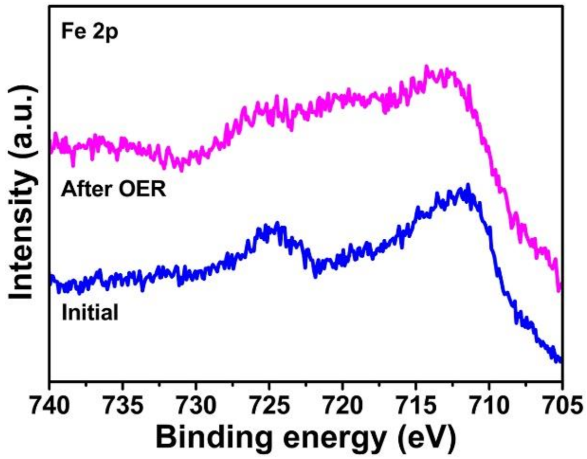

Figure S12. Fe 2p XPS spectrum of the NiFe-BDC-0.5 before and after OER.

Table S1. Comparisons of the OER performances of the NiFe-BDC-0.5 with other reported transition metal-based MOFs in the literatures in 1 M KOH.

Catalyst	η_{10} (mV)	Tafel Slope (mV dec^{-1})	Reference
**NiFe-BDC-0.5**	**256**	**45.4**	**This work**
NiFe-MOF	215	49.1	Adv. Funct. Mater., 2021, 31, 2102066 [60]
HO_{oct}-$NiFe_2O_4$/IF	260	36.1	Adv. Funct. Mater., 2022, 32, 2201011 [27]
Ni/$NiFe_2O_4$@PPy	265	99	Chem. Eng. J., 2023, 454, 140278 [61]
NiCo-LDH-TPA	267	52.4	Angew. Chem. Int. Ed., 2021, 60, 10614-10619 [62]
NiS/NiO@N-C	269	48.4	Chem. Eng. J., 2022, 428, 131094 [63]
NiOOH/FeOOH NB	270	41	J. Mater. Chem. A, 2021, 9, 15586-15594 [64]
CoP-NC@NiFeP	270	84	Chem. Eng. J., 2022, 428, 131115 [28]
NiFeS-NS	273	49	Chin Chem Lett., 2022, 33, 3916-3920 [65]
CoFe-MOFs	274	46.7	J. Mater. Chem. A, 2020, 8, 190 [66]
MCCF/NiMn-MOFs	280	86	Angew. Chem. Int. Ed., 2020, 59, 18234-18239 [67]
NiFe-Se/CFP	281	40.93	ACS Sustainable Chem. Eng., 2021, 9, 2047-2056 [68]
FeNi/$NiFe_2O_4$	283	46.5	Chem. Sci., 2022, 13, 9440-9449 [69]

**Catalyst**	**η_{10}（mV）**	**Tafel Slope（mV dec^{-1}）**	**Reference**
**NiFe-BDC-0.5**	**256**	**45.4**	**This work**
LiS@NiFe-LDH	286	50.73	Energy Environ. Sci., 2020, 13, 1711-1716 [71]
$FeNi_2P$-NPs	286	70	Catal. Sci. Technol., 2023, 13, 1512-1517 [72]
CoZn-MOFs	287	76.3	Small, 2021, 17, 2105150 [73]
$Co_{0.7}Fe_{0.3}CB$	295	36.2	Adv. Funct. Mater., 2020, 30, 1909889 [74]
P-CoPc@CNT	300	41.7	Adv. Sci., 2023, 10, 2206107 [75]
Br-Ni-MOF	306	79.1	Sci. Adv., 2021, 7, eabk0919 [76]
FeNiCo@NC-P	310	64	Adv. Funct. Mater., 2020, 30, 1908167 [77]
W_2N/WC	320	94.5	Adv. Mater., 2020, 32, 1905679 [78]
Fe_3C-Co/NC	340	-	Adv. Funct. Mater., 2019, 29, 1901949 [79]
FeNi/N-CPCF	355	67	Appl. Catal. B-Environ., 2020, 263, 118344 [29]
Fe_2Ni-BPTC	365	81.8	Angew. Chem. Int. Ed., 2018, 57, 9660-9664 [80]

Table S2. The contents of Ni, Fe, C, and O in NiFe-BDC-0.5 before and after OER test.

Element	Content	
	Initial (at.%)	After OER (at.%)
Ni	2.98	10.98
Fe	1.21	4.20
C	55.30	37.55
O	40.52	45.87
